# Downregulation of Drp1 and Fis1 Inhibits Mitochondrial Fission and Prevents High Glucose-Induced Apoptosis in Retinal Endothelial Cells

**DOI:** 10.3390/cells9071662

**Published:** 2020-07-10

**Authors:** Dongjoon Kim, Aravind Sankaramoorthy, Sayon Roy

**Affiliations:** 1 Department of Medicine, Boston University School of Medicine, Boston, MA 02118, USA; djkim@bu.edu (D.K.); asankara@bu.edu (A.S.); 2 Department of Ophthalmology, Boston University School of Medicine, Boston, MA 02118, USA

**Keywords:** mitochondria, high glucose, fission, retinal endothelial cells, diabetic retinopathy

## Abstract

Diabetic retinopathy is a prevalent microvascular complication characterized by apoptotic vascular cell loss in the retina. Previous studies have shown that high glucose (HG)-induced mitochondrial fragmentation plays a critical role in promoting retinal vascular cell apoptosis. Here, we investigated whether downregulation of mitochondrial fission genes, Fis1 and Drp1, which are overexpressed in HG condition, prevents mitochondrial fragmentation, preserves mitochondrial function, and protects retinal endothelial cells from apoptosis. Rat retinal endothelial cells (RRECs) were grown in normal (5 mM glucose) or HG (30 mM glucose) medium; in parallel, cells grown in HG medium were transfected with either Fis1 siRNA or Drp1 siRNA, or both siRNAs in combination, or scrambled siRNA as control. Live-cell confocal imaging showed decreased mitochondrial fission in cells transfected with Fis1 siRNA or Drp1 siRNA concomitant with reduced TUNEL-positive cells and a decrease in the expression of pro-apoptotic proteins, Bax and cleaved caspase 3, under HG condition. Importantly, the combined siRNA approach against Fis1 and Drp1 prevented HG-induced changes in the oxygen consumption rate (OCR) and extracellular acidification rate (ECAR). The findings from this study indicate that reducing HG-induced overexpression of mitochondrial fission genes preserves mitochondrial morphology and prevents retinal vascular cell apoptosis associated with diabetic retinopathy.

## 1. Introduction

Early retinal vascular lesions involving apoptotic cell death represent a prominent characteristic of diabetic retinopathy, the leading cause of blindness in the working age population [1,2,3,4,5,6,7,8]. Studies suggest changes in mitochondrial morphology can compromise mitochondrial functionality, as evidenced by altered mitochondrial membrane potential heterogeneity and reduced oxygen consumption concomitant with excess mitochondrial fragmentation [9,10,11]. Our previous work demonstrated that the HG condition induces mitochondrial fragmentation in retinal vascular cells, and thereby contributes to mitochondrial dysfunction [10,11]. However, the link between HG-induced mitochondrial morphological changes and its effect on mitochondrial function leading to apoptosis is currently not well understood.

Fusion and fission are cellular events that participate in the maintenance of mitochondrial morphology and its network dynamics [9]. Mitochondrial fission is balanced by opposing fusion events [12]; however, a disruption of the delicate balance between the two events could promote changes in mitochondrial morphology, including fragmentation, that may contribute to apoptotic cell death [13]. Drp1 (Dynamin-related protein 1), the master regulator of mitochondrial fission, is a cytosolic member of the dynamin family of GTPases [14,15]. It polymerizes around the mitochondrion and, through GTP hydrolysis, promotes fission and ultimately fragmentation. Moreover, Drp1 binding to mitochondria increases during apoptosis [16]. Additionally, Fis1, a mitochondrial outer membrane protein and an important regulator of mitochondrial dynamics, mediates the assembly of mitochondrial fission complexes involved in the execution of mitochondrial fragmentation [17,18].

Fis1 is involved in promoting apoptosis through the interaction with endoplasmic reticulum-localized Bap31 in Bax/Bak-mediated permeabilization of mitochondrial outer membrane, leading to cytochrome c release and ultimately cell death [19]. In addition, Fis1 has been implicated in inhibiting the GTPase activity of fusion proteins Mfn1, Mfn2, and Opa1 [20]. In contrast, Fis1 is not known to interfere with the GTPase activity of Drp1 [20]. Of note, downregulation of mitochondrial fusion genes, Mfn1, Mfn2, and Opa1, in concert with upregulation of mitochondrial fission genes, Drp1 and Fis1, has been reported to facilitate mitochondrial fragmentation [21]. Additional studies indicate that Mfn1-deficient cells exhibit small mitochondrial fragments, while Mfn2-deficient cells exhibit larger mitochondrial fragments [22,23], suggesting that impaired mitochondrial fusion not only contributes to mitochondrial fragmentation, but may influence the size of the mitochondrial fragments. While decreased Mfn1, Mfn2 [24], Opa1, and other fusion genes can influence mitochondrial fragmentation, Fis1 and Drp1 upregulation can directly promote mitochondrial fragmentation [25]. Importantly, Fis1 and Drp1 downregulation has been shown to inhibit cell death [25]. Although abnormal levels of fission activity are known to compromise cellular metabolism, the involvement of these two fission genes in HG-induced metabolic changes and apoptotic cell death associated with diabetic retinopathy is not well understood.

Increased mitochondrial fission and its deleterious consequences leading to mitochondrial fragmentation and promoting HG-induced apoptosis have been well established. However, it is unknown if suppressing excess mitochondrial fragmentation through modulation of fission gene activity shows beneficial effects in preventing apoptotic cell death associated with diabetic retinopathy. Therefore, the present study investigated whether reducing HG-induced abnormal Drp1 and Fis1 overexpression using a siRNA approach could ameliorate mitochondrial fragmentation, improve mitochondrial metabolic function, and thereby prevent apoptosis in retinal endothelial cells.

## 2. Materials and Methods

### 2.1. Cell Culture

RRECs isolated from rat retinas and verified for presence of Von Willebrand factor were used in the present study. Experiments conducted in the present study utilized different preparations of retinal endothelial cells. To assess the effects of HG on mitochondrial fission proteins, RRECs were cultured in normal (N, 5 mM D-glucose) or high glucose (HG, 30 mM) Dulbecco’s modified Eagle’s medium (DMEM) supplemented with 10% fetal bovine serum (FBS) and 1× antimycotic/antibiotic mixture for 7 days. To investigate the effect of downregulating HG-induced overexpression of mitochondrial fission genes on apoptosis, cells were grown in high glucose medium and transfected with Fis1 siRNA or Drp1 siRNA (Ambion) individually, with both siRNAs in combination, or with scrambled siRNA (Qiagen), using Lipofectin reagent. Total cell lysate was extracted using 200 µL of radioimmunoprecipitation assay (RIPA) lysis buffer containing protease inhibitors (Santa Cruz Biotechnology) followed by incubation in ice for 15 min and centrifugation at 700× *g* for 5 min at 4 ℃. Amount of protein was evaluated using the bicinchoninic acid (BCA) protein assay (Pierce Chemical, Rockford, IL, USA).

### 2.2. Western Blot Analysis

To determine the effect of HG on the mitochondrial fission protein expression and to further investigate the effect of downregulation of HG-induced increased mitochondrial fission protein expression on apoptosis, 20 µg of the total proteins was separated using 12% SDS-PAGE and transferred onto a polyvinylidene difluoride (PVDF) membrane (Millipore, Billerica, MA, USA) using a semidry apparatus (Bio-Rad) and blocked with 5% non-fat dry milk for 1 h and incubated overnight at 4 °C with antibodies against Fis1 (1:1000, Catalog No. PA1-41082; Thermo Scientific), Drp1 (1:1000, Catalog No. sc-271583; Santacruz), cleaved caspase-3 (1:1000, Catalog No. 9661; Cell Signaling), or Bax antibody (1:1000, Catalog No. 2772; Cell Signaling) in a solution of Tris-buffered saline with 0.1% Tween-20 (TTBS) and 5% BSA. The next day, the membranes underwent several washes with TTBS and were exposed to a secondary antibody solution with AP-conjugated anti-rabbit IgG (1:3000, Catalog No. 7054; Cell Signaling) or AP-conjugated anti-mouse IgG (1:3000, Catalog No. 7056; Cell Signaling) corresponding to the appropriate primary antibodies for 1 h in room temperature. The membranes were then washed with TTBS and subjected to Immun-Star chemiluminescent AP substrate (Bio-Rad) and imaged using chemiluminescence imager (Fuji film LAS-4000, Tokyo, Japan). Equal loading of proteins was verified by β-actin antibody (1:1000, Catalog No. 4967; Cell Signaling) and by Ponceau-S staining following transfer. Densitometric analysis was performed to quantify changes in protein expression at non-saturating exposures and analyzed using the ImageJ software (National Institutes of Health, Bethesda, MD, USA).

### 2.3. Live Confocal Microscopy and Analysis of Mitochondrial Morphology

Live confocal imaging was performed on cells from each experimental group to evaluate the mitochondrial morphology by Zeiss LSM 510 Meta microscope (Carl Zeiss, Oberkochen, Germany) using a 63× oil immersion objective. During the imaging process, cells were maintained at 37 °C in a 5% CO_2_ humidified microscope stage chamber. Cells stained with MitoTracker Red (250 nM; Molecular Probes, Eugene, OR, USA) for 45 min were exposed to helium/neon laser excitation at 543 nm and emission through a bandpass filter ranging from 650 to 710 nm. Mitochondrial morphology was analyzed using ImageJ software (NIH) by assessing values of form factor (FF; (4π*Area/perimeter^2^)) and aspect ratio (AR; ratio of lengths of major and minor axes), as described previously [26,27]. AR is indicative of mitochondrial length, where an AR value of 1 represents a perfect circle, while an AR value increases as mitochondria becomes elongated and more elliptical. FF is indicative of both mitochondrial length and degree of mitochondrial branching, where an FF value of 1 represents a circular mitochondrion with no branching, while a higher FF value signifies a longer mitochondrion with a higher degree of branching.

### 2.4. Seahorse Analysis of Oxygen Consumption and Extracellular Acidification Rates

To assess the effect of reducing fission genes on mitochondrial metabolic activity, the rates of oxygen consumption and extracellular acidification were obtained using a bioenergetic assay (XFe96; Seahorse Bioscience, Billerica, MA, USA). Cells cultured in a 96-well microplate were subjected to XF assay medium in a CO_2_-free incubator at 37 °C for 1 h in order to reach equilibrium in temperature and pH levels. To measure the oxygen consumption rate (OCR), steady-state OCR was initially obtained at the fifth time point, followed by injections of oligomycin (5 μM), an inhibitor of ATP synthase, and FCCP (carbonyl cyanide-4-[trifluoromethoxy] phenylhydrazone, 1 μM), an uncoupler of mitochondrial oxidative phosphorylation, to assess the maximal OCR. Subsequently, injection of Antimycin A (a complex III inhibitor, 5 μM) was carried out to confirm that previously observed changes in OCR were driven by mitochondrial respiration. To measure the extracellular acidification rate (ECAR), steady-state ECAR was obtained at the fifth time point, followed by injections of glucose to obtain the basal glycolytic rate, and oligomycin to inhibit ATP synthase to assess glycolytic capacity. Lastly, injection of 2-deoxyglucose, a glucose analogue, was carried out to inhibit glycolysis and to verify that previously observed changes in ECAR were driven by glycolysis.

### 2.5. TUNEL Assay

To assess the effect of reducing overexpression of mitochondrial fission genes on apoptotic cell death, cells from all experimental groups were subjected to TUNEL (Terminal dUTP Nick-End Labeling) assay using the ApopTag Fluorescein Direct in Situ Apoptosis Detection Kit (Millipore, Billerica, MA, USA), following instructions provided by the manufacturer. Fixation of RRECs cultured on coverslips was performed briefly with 4% paraformaldehyde, followed by exposure to a pre-cooled solution of ethanol/acetic acid at a 2:1 ratio. Cells underwent two phosphate-buffered saline (PBS) washes followed by incubations with the equilibrium buffer and TdT enzyme in a moist chamber at 37 °C for 1 h. Following incubation, cells underwent further PBS washes and were exposed to a solution containing anti-digoxigenin peroxidase. Cells were stained with 4’,6-diamidino-2-phenylindole (DAPI), mounted with an anti-fade reagent (SlowFade; Molecular Probes, Eugene, OR, USA), and ten random fields from each coverslip were imaged for analysis through a digital microscope (DS-Fi1; Nikon, Tokyo, Japan).

### 2.6. Statistical Analysis

Data are shown as mean ± standard deviation (SD). Control group values are normalized to 100%, while other experimental group values are represented as percentages of control. Statistical analysis was performed using the normalized values. One-way analysis of variance (ANOVA) followed by Bonferroni’s post-hoc test was carried out to perform comparisons between groups. *p* < 0.05 was considered statistically significant.

## 3. Results

### 3.1. Effects of HG and Drp1/Fis1 siRNAs on Fission Genes in RRECs

**Figure 1:**
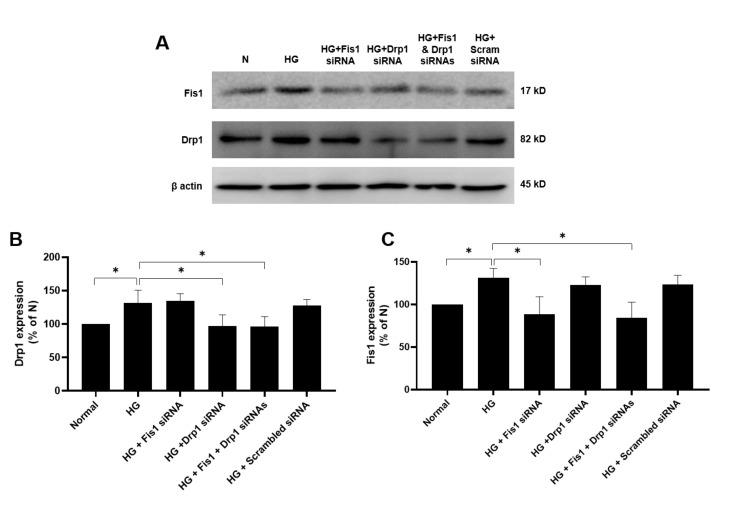
Effect of Fis1/Drp1 siRNA on high glucose (HG)-induced overexpression of Fis1 and Drp1 expression in rat retinal endothelial cells (RRECs). (**A**) Representative Western blot (WB) image shows Fis1 and Drp1 expression is significantly upregulated in cells grown in HG medium. In parallel, cells grown in HG and transfected with Fis1 siRNA, Drp1 siRNA, or both siRNAs showed corresponding decreases in Fis1 or Drp1 expression. Graphical illustration of cumulative data shows Drp1 (**B**) and Fis1 (**C**) are significantly upregulated in cells grown in HG condition and reduced in cells transfected with Drp1 and Fis1 siRNAs. Data are expressed as mean ± SD. * *p* < 0.05. *n* = 6.

Cells grown in HG medium exhibited significant upregulation of Drp1 (135 ± 11% of N; *p* < 0.05, Figure 1A,B) and Fis1 (131 ± 11% of N; *p* < 0.05, Figure 1A,C) compared with cells grown in N medium. Cells grown in HG medium transfected with Drp1 siRNA or a combination of both Drp1 siRNA and Fis1 siRNA showed a reduction in Drp1 gene expression (97 ± 17% of N; *p* < 0.05; 96 ±15% of N; *p* < 0.05, respectively, Figure 1A,B) compared with that of cells grown in HG. In parallel, cells grown in HG medium transfected with Fis1 siRNA or a combination of both Drp1 siRNA and Fis1 siRNA showed Fis1 downregulation (88 ± 21% of N; *p* < 0.05, 84 ± 19% of N; *p* < 0.05, respectively, Figure 1A,C) compared with that of cells grown in HG. Cells grown in HG medium and transfected with scrambled siRNA used as negative control showed no significant difference in Drp1 (128 ± 9% of N; *p* > 0.05; Figure 1A,B) or Fis1 (123 ± 11% of N; *p* > 0.05; Figure 1A,C) expression compared with cells grown in HG medium alone.

### 3.2. Effects of HG and Drp1/Fis1 siRNAs on Apoptotic Genes in RRECs

**Figure 2:**
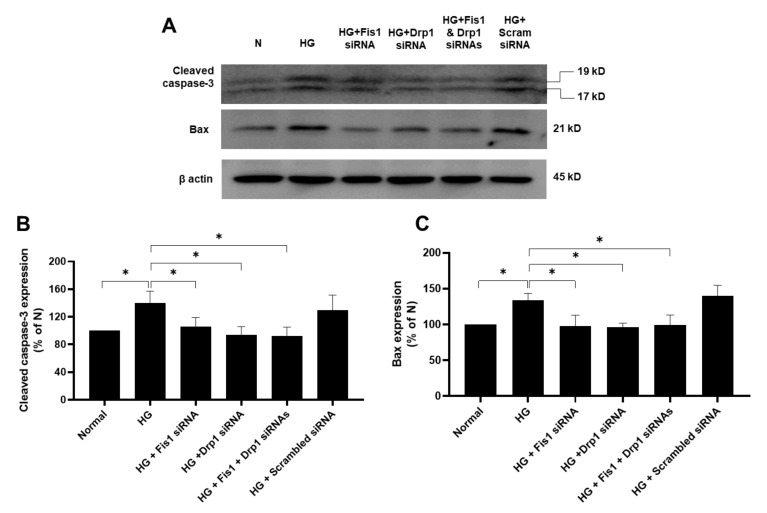
Effect of reducing HG-induced overexpression of fission genes on apoptosis. (**A**) Representative WB image shows cleaved caspase-3 and Bax expression are significantly upregulated in cells grown in HG medium. In contrast, cells grown in HG medium and transfected with Fis1 siRNA, Drp1 siRNA, or both siRNAs exhibit reduced levels of these pro-apoptotic genes. Graphical illustration of cumulative data shows (**B**) cleaved caspase-3 and (**C**) Bax levels are downregulated in cells transfected with Fis1 siRNA, Drp1 siRNA, or both siRNAs compared with those of cells grown in HG medium alone. Data are expressed as mean ± SD. * *p* < 0.05. *n* = 6.

RRECs grown in HG medium had significantly increased levels of cleaved caspase-3 (140 ± 17% of N; *p* < 0.05; Figure 2A,B) and Bax (134 ± 10% of N; *p* < 0.05, Figure 2A,C) compared with those of cells grown in N medium. Interestingly, cells grown in HG medium transfected with Fis1 siRNA, Drp1 siRNA, or a combination of both Drp1 siRNA and Fis1 siRNA showed a reduction in cleaved caspase-3 (106 ± 13% of N; *p* < 0.05, 97 ± 12% of N; *p* < 0.05, 92 ± 13% of N; *p* < 0.05; respectively, Figure 2A,B) or Bax expression (98 ± 15% of N; *p* < 0.05, 98 ± 6% of N; *p* < 0.05, 99 ± 14% of N; *p* < 0.05; respectively, Figure 2A,C) compared with those of cells grown in HG. Cells grown in HG medium and transfected with scrambled siRNA used as negative control showed no significant difference in cleaved caspase-3 (129 ± 22; *p* > 0.05; Figure 2A,B) or Bax expression (140 ± 14% of N; *p* > 0.05; Figure 2A,C) compared with those of cells grown in HG medium alone.

### 3.3. Drp1/Fis1 Downregulation Prevents Mitochondrial Fragmentation in RRECs

**Figure 3:**
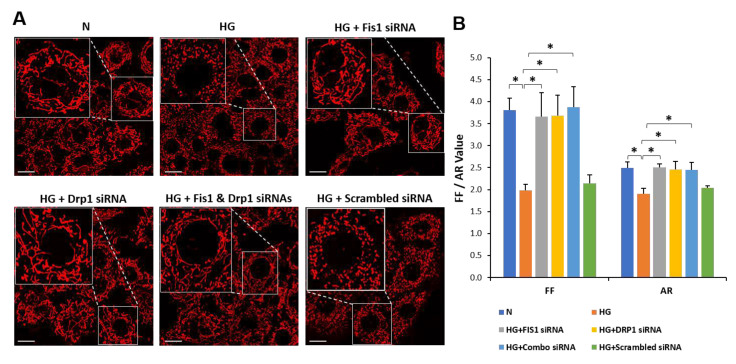
Reducing fission protects against HG-induced mitochondrial fragmentation. (**A**) Representative images of mitochondrial networks of RRECs grown in normal medium, HG, HG transfected with DRP1 siRNA, HG transfected with FIS1 siRNA, HG transfected with DRP1 siRNA and FIS 1 siRNA (Combo siRNA), or HG transfected with scrambled siRNA. Cells grown in HG medium or HG medium transfected with scrambled siRNA show fragmented mitochondrial networks, while cells grown in N medium or cells transfected with siRNAs exhibit tubular, elongated mitochondrial morphology. The large inset represents an enlarged view of the corresponding image (indicated by dotted lines) in the small inset in each panel. Scale bar = 20 μm. (**B**) Graphical illustration of cumulative data shows that reducing fission genes, Drp1 and Fis1, significantly prevents HG-induced mitochondrial fragmentation. Data are expressed as mean ± SD. * *p* < 0.05. *n* = 6. FF, form factor; AR, aspect ratio.

Live mitochondrial imaging data using confocal microscopy indicated that cells grown in HG medium had a significant decrease in FF and AR values compared with those of cells grown in N medium (HG: FF = 1.99 ± 0.13; *p* < 0.01; AR = 1.91 ± 0.12; *p* < 0.01, Figure 3A,B; N: FF = 3.80 ± 0.27. AR = 2.50 ± 0.14, Figure 3A,B). Interestingly, cells grown in HG medium and transfected with Drp1 siRNA, Fis1 siRNA, or a combination of both Drp1 siRNA and Fis1 siRNA exhibited significant increase in the AR and FF values (HG + Drp1 siRNA: FF = 3.68 ± 0.46; *p* < 0.01; AR = 2.46 ± 0.18; *p* < 0.01, Figure 3A,B; HG + Fis1 siRNA: FF = 3.66 ± 0.55; *p* < 0.01; AR = 2.51 ± 0.08; *p* < 0.01, Figure 3A,B; HG + combo siRNA: FF = 3.88 ± 0.46; *p* < 0.01; AR = 2.44 ± 0.17; *p* < 0.01, Figure 3A,B) compared with cells grown in HG medium alone. Cells grown in HG medium and transfected with scrambled siRNA used as negative control showed no significant difference in FF or AR values compared with cells grown in HG medium (HG + scrambled siRNA: FF = 2.14 ± 0.19; *p* > 0.05; AR = 2.04 ± 0.05; *p* > 0.05, Figure 3A,B).

### 3.4. Reduced Mitochondrial Fission Prevents HG-Induced Apoptosis in RRECs

**Figure 4:**
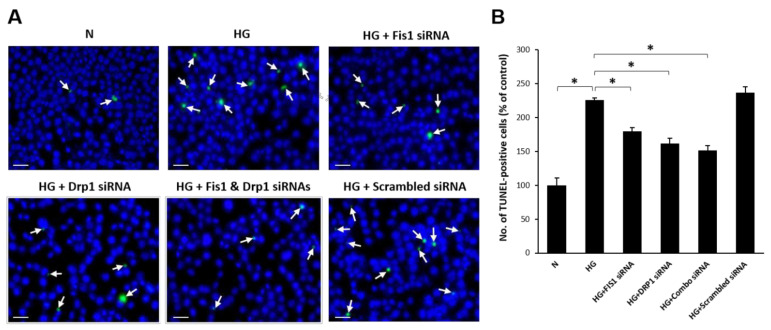
Reducing fission protects against HG-induced apoptosis. (**A**) Representative images of TUNEL-positive RRECs (white arrows) grown in normal medium, HG, HG transfected with DRP1 siRNA, HG transfected with FIS1 siRNA, HG transfected with DRP1 siRNA and FIS 1 siRNA, or HG transfected with scrambled siRNA. Scale bar = 50 μm. (**B**) Graphical illustration of cumulative data indicates that cells grown in HG medium or HG medium transfected with scrambled siRNA exhibit an increased number of TUNEL-positive cells. In parallel, downregulation of DRP1 and FIS1 fission genes significantly protects against HG-induced apoptotic cell death. Data are expressed as mean ± SD. * *p* < 0.05. *n* = 6.

TUNEL data showed that cells grown in HG medium had a significant increase in the number of apoptotic cells compared with cells grown in N medium (48.6 ± 2.9% of N; *p* < 0.01; Figure 4A,B). Interestingly, cells grown in HG medium transfected with Drp1 siRNA (48.6 ± 2.9% of N; *p* < 0.01; Figure 4A,B), Fis1 siRNA (48.6 ± 2.9% of N; *p* < 0.01; Figure 4A,B), or a combination of both Drp1 siRNA and Fis1 siRNA (48.6 ± 2.9% of N; *p* < 0.01; Figure 4A,B) exhibited a significant decrease in the number of apoptotic cells compared with cells grown in HG medium alone. Cells grown in HG medium transfected with scrambled siRNA (48.6 ± 2.9% of N; *p* < 0.01; Figure 4A,B) exhibited no significant change in the number of apoptotic cells compared with cells grown in HG medium.

### 3.5. Inhibiting Mitochondrial Fission Gene Expression Improves Mitochondrial Respiration in RRECs

**Figure 5:**
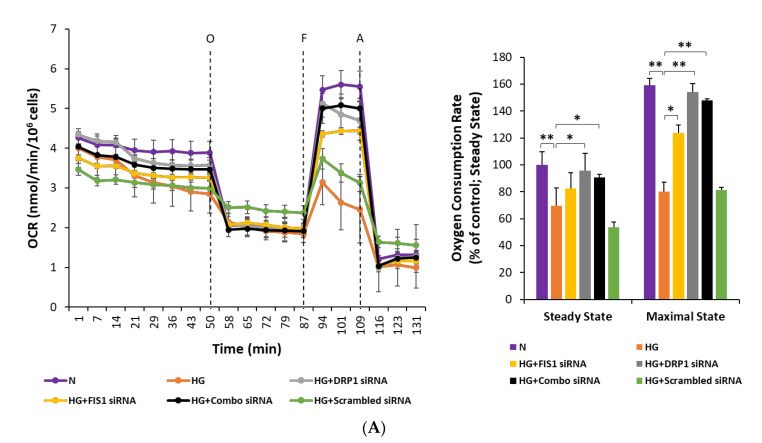

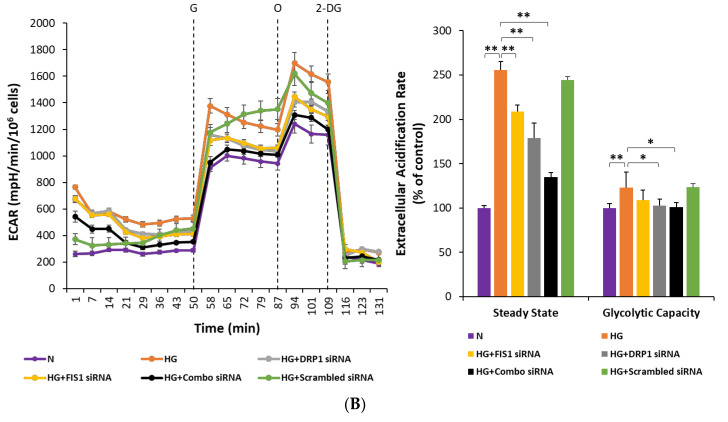
(**A**) Reducing mitochondrial fission protects against HG-induced decrease in oxygen consumption rate (OCR) in RRECs. At the fifth time point, steady-state oxygen consumption was measured. Subsequently, oligomycin was injected (vertical line O) to inhibit ATP synthase, and carbonyl cyanide-4-[trifluoromethoxy] phenylhydrazone (FCCP) was injected (vertical line F) to uncouple mitochondria and obtain maximal oxygen consumption rate at the eleventh time point. Antimycin A was injected (vertical line A) to confirm that the respiration changes were primarily owing to mitochondrial respiration. The cumulative OCR data are represented by the bar graph that shows RRECs grown in N media, HG media, HG media transfected with DRP1 siRNA, HG media transfected with FIS1 siRNA, HG media transfected with both DRP1 siRNA and FIS1 siRNA (Combo siRNA), or HG transfected with scrambled siRNA. Data are expressed as mean ± SD. * *p* < 0.05, ** *p* < 0.01. *n* = 6. (**B**) Reducing mitochondrial fission protects against HG-induced increase in extracellular acidification (ECAR) in RRECs. Steady-state extracellular acidification rate was measured at the fifth time point. This was followed by the injection of glucose (vertical line G) to trigger glycolysis under basal conditions, and oligomycin was injected (vertical line O) to inhibit ATP synthase to assess glycolytic capacity at the eleventh time point. 2-deoxyglucose, a glucose analogue, was then injected (vertical line 2-DG) to inhibit glycolysis and to confirm that the respiration changes were primarily owing to glycolysis. The cumulative ECAR data are represented by the bar graph that shows RRECs grown in normal medium, HG, HG transfected with DRP1 siRNA, HG transfected with FIS1 siRNA, HG media transfected with both DRP1 siRNA and FIS1 siRNA (Combo siRNA), or HG transfected with scrambled siRNA. Lines G, O, and 2-DG indicate injections of glucose, oligomycin, and 2-deoxyglucose, respectively. Data are expressed as mean ± SD. * *p* < 0.05, ** *p* < 0.01. *n* = 6.

To determine the effects of reducing the expression of HG-induced fission genes on metabolic capacity and extracellular acidification, simultaneous measurements for rates of cellular oxygen consumption and extracellular acidification were carried out in RRECs grown in N medium, HG medium, HG transfected with Fis1 siRNA, HG transfected with Drp1 siRNA, HG transfected with both Fis1 and Drp1 siRNAs, and HG transfected with scrambled siRNA using XF96e bioenergetic assay. For measurement of the oxygen consumption rate, oligomycin (vertical line O; Figure 5A) was injected to inhibit ATP synthase, followed by injection of FCCP (vertical line F; Figure 5A) to uncouple mitochondria. Finally, Antimycin A was injected (vertical line A; Figure 5A) to confirm that the respiration changes were primarily owing to mitochondrial respiration. For measurement of extracellular acidification rate, glucose was injected (vertical line G; Figure 5B) to obtain the rate of glycolysis under basal conditions, followed by addition of oligomycin (vertical line O; Figure 5B) to inhibit ATP synthase to assess glycolytic capacity. Finally, injection of 2-deoxyglucose, a glucose analogue, was carried out (vertical line 2-DG; Figure 5B) to inhibit glycolysis and to confirm that the respiration changes were mainly owing to glycolysis.

RRECs grown in HG medium exhibited a significant decrease of steady state and maximal oxygen consumption (Figure 5A; steady state: 70 ± 13% of N; vs. 100 ± 10% of N in normal, *p* < 0.01, *n* = 5; maximal: 80 ± 7% of N; vs. 159 ± 5% of N in normal, *p* < 0.01, *n* = 5) compared with those of cells grown in normal medium. Cells grown in HG medium and transfected with Fis1 siRNA (Figure 5A; steady state: 83 ± 12% of N; *p* > 0.05, *n* = 5, maximal: 124 ± 6% of N; *p* < 0.05, *n* = 5), or Drp1 siRNA (Figure 5A; steady state: 96 ± 13% of N; *p* < 0.05, *n* = 5, maximal: 154 ± 6% of N; *p* < 0.01, *n* = 5), or both Fis1 and Drp1 siRNAs (Figure 5A; steady state: 91 ± 2% of N; *p* < 0.05, *n* = 5, maximal: 148 ± 1% of N; *p* < 0.01, *n* = 5) showed improvement in oxygen consumption rates compared with those of cells grown in HG medium alone. Cells grown in HG medium and transfected with scrambled siRNA showed no significant change in oxygen consumption rate (Figure 5A; steady state: 53 ± 4% of N; *p* > 0.01, *n* = 5, maximal: 81 ± 2% of N; *p* > 0.01, *n* = 5) compared with that of cells grown in HG medium.

To determine whether reducing HG-induced fission gene overexpression mitigates glycolytic rates, extracellular acidification rates were assessed in the experimental groups. RRECs grown in HG showed a significant increase in the steady-state rate of extracellular acidification and glycolytic capacity (Figure 5B; steady state: 256 ± 9% of N; vs. 100 ± 3% of N in normal, *p* < 0.01, *n* = 5; glycolytic capacity: 123 ± 17% of N; vs. 100 ± 5% of N in normal, *p* < 0.01, *n* = 5). Cells grown in HG medium and transfected with Fis1 siRNA (Figure 5B; steady state: 209 ± 8% of N; *p* < 0.01, *n* = 5, glycolytic capacity: 109 ± 11% of N; *p* > 0.05, *n* = 5), or Drp1 siRNA (Figure 5B; steady state: 179 ± 17% of N; *p* < 0.01, *n* = 5, glycolytic capacity: 103 ± 7% of N; *p* < 0.05, *n* = 5), or both Fis1 and Drp1 siRNAs (Figure 5B; steady state: 135 ± 5% of N; *p* < 0.01, *n* = 5, glycolytic capacity: 101 ± 5% of N; *p* < 0.05, *n* = 5) showed improvement in steady-state extracellular acidification rate and glycolytic capacity compared with those of cells grown in HG medium alone. Cells grown in HG medium and transfected with scrambled siRNA showed no significant change in steady-state extracellular acidification rate or glycolytic capacity (Figure 5B; steady state: 245 ± 3% of N; *p* > 0.01, *n* = 5, glycolytic capacity: 124 ± 4% of N; *p* > 0.01, *n* = 5) compared with that of cells grown in HG medium.

## 4. Discussion

In this study, we present evidence that shows reducing HG-induced fission gene overexpression and inhibiting mitochondrial fission activity prevents mitochondrial fragmentation and restores mitochondrial functionality. Our findings indicate that RRECs grown in HG upregulate Drp1 and Fis1 expression and promote mitochondrial fragmentation concomitant with increased cleaved caspase-3, Bax activity, impaired mitochondrial respiration, and apoptosis. Reducing HG-induced Drp1 or Fis1 overexpression using a siRNA approach reversed the aforementioned deleterious effects and prevented HG-induced apoptosis associated with diabetic retinopathy.

Fis1 and Drp1 overexpression has been reported to play a significant role in the pathogenesis of diabetic microangiopathies. Studies show that Drp1 upregulation leads to increased mitochondrial fragmentation in glomerular podocytes of patients with diabetic nephropathy [28], suggesting that Drp1-mediated mitochondrial fission can contribute to this disease process. Podocyte-specific deletion of Drp1 in diabetic mice resulted in preservation of mitochondrial structure, protection against albuminuria, and improvement in mesangial matrix expansion and podocyte morphology [29]. Similarly, Drp1-mediated mitochondrial fission was found to be enhanced in the myocardium of diabetic mice, and that Drp1 inhibition decreased mitochondrial fission and protected against myocardial ischemia/reperfusion injury in diabetic mice [30]. Interestingly, a study reported that diabetes-induced synaptic impairment in the hippocampus of mice can be protected by inhibiting GSK3β, an upstream signaling molecule that upregulates Drp1 activity in diabetic conditions [31], suggesting that reducing Drp1 activity may have beneficial effects. In aortic venous endothelial cells isolated from diabetic patients, Fis1 expression and mitochondrial fragmentation were significantly upregulated [32]. Using siRNA-mediated Fis1 downregulation, HG-induced mitochondrial fission and reactive oxygen species (ROS) production were prevented [32]. Taken together, these findings demonstrate that abnormal Fis1 and Drp1 upregulation can promote mitochondrial dysfunction and contribute to the pathogenesis of diabetic microangiopathies.

The results from the current study have shed light on the relative contributory role of Drp1 and Fis1 in mitochondrial fragmentation related to vascular cell loss in diabetic retinopathy. Data suggest both Drp1 and Fis1 are upregulated under HG condition and contribute to mitochondrial fragmentation. Drp1 downregulation appeared to be more effective than Fis1 downregulation in reducing the number of TUNEL-positive cells. Importantly, downregulating both Drp1 and Fis1 using a combination siRNA approach produced a robust effect as opposed to targeting either of the two genes independently. A higher level of efficacy from combined siRNA approach has been previously reported by other studies in which reducing both Fis1 and Drp1 led to an additive effect [33]. We have also observed that combined antisense oligonucleotides targeting multiple extracellular matrix genes was more effective than targeting a single gene in reducing vascular basement membrane thickening associated with diabetic retinopathy [34]. Findings from the current study suggest that a combined siRNA approach is likely to be more efficacious in preventing HG-induced mitochondrial fragmentation, rescuing cells from HG-induced apoptosis, and improving metabolic functions in HG milieu.

Mitophagy activation is known to induce the metabolic switch in metastatic and embryogenic processes [35,36,37,38], and mitophagy can induce the metabolic switch to either oxidative phosphorylation or glycolysis depending on energy demands [39]. In fibroblasts, mitophagy is not activated even though the metabolic switch from oxidative phosphorylation to glycolysis occurs [40]. Taken together, these findings suggest that further studies are necessary to determine the effects of HG on the metabolic switch in the context of mitophagy.

In the current study, in vitro findings indicate HG-induced upregulation of Drp1 and Fis1 can lead to increased apoptosis of retinal vascular cells. However, these findings will need to be verified in animal models of diabetic retinopathy. Currently, there are efforts attempting to develop “mitochondrial medicine” in which mitochondrial dysfunction is targeted. Although the role of mitochondrial dysfunction has been established in human diseases and is widely recognized, there is a lack of clinical trials testing strategies for ameliorating mitochondrial damage. Under ClinicalTrials.gov, a study examining the effects of hyperglycemia on mitochondrial fragmentation in human arterial endothelial cells derived through J-wire biopsy is currently underway (ClinicalTrials.gov Identifier: NCT02682342). Further studies are needed to determine whether targeting mitochondrial fission genes provide beneficial effects in vivo.

While Drp1 and Fis1 play critical roles in regulating mitochondrial structure, other genes involved in mitochondrial dynamics may also participate in this process. Further studies are necessary to investigate other fission/fusion proteins and their roles in regulating mitochondrial morphology in HG condition. To the best of our knowledge, the present study demonstrates for the first time that reducing HG-induced abnormal Drp1 and Fis1 overexpression using a combined siRNA approach is effective in preventing mitochondrial fragmentation, improving mitochondrial respiration, and inhibiting apoptosis in retinal endothelial cells. Taken together, the findings from this study indicate that targeting mitochondrial fission gene overexpression may be beneficial in reducing vascular lesions associated with diabetic retinopathy.
